# A Deployment of Fine-Grained Sensor Network and Empirical Analysis of Urban Temperature

**DOI:** 10.3390/s100302217

**Published:** 2010-03-18

**Authors:** Niwat Thepvilojanapong, Takahiro Ono, Yoshito Tobe

**Affiliations:** 1 School of Science and Technology for Future Life, Tokyo Denki University, 501 Daikou Building, 2–5 Kanda-Nishikichou, Chiyoda, Tokyo, 101–0054, Japan; 2 Core Research for Evolutional Science and Technology, Japan Science and Technology Agency, Japan; E-Mail: yoshito tobe@osoite.jp; 3 Department of Information and Media Engineering, Tokyo Denki University, 2–2 Kanda-Nishikichou, Chiyoda, Tokyo, 101–8457, Japan; E-Mail: tak@u-netlab.jp

**Keywords:** urban sensing, fine-grained sensor network, fine granularity, temperature, empirical analysis, clustering

## Abstract

Temperature in an urban area exhibits a complicated pattern due to complexity of infrastructure. Despite geographical proximity, structures of a group of buildings and streets affect changes in temperature. To investigate the pattern of fine-grained distribution of temperature, we installed a densely distributed sensor network called *UScan*. In this paper, we describe the system architecture of UScan as well as experience learned from installing 200 sensors in downtown Tokyo. The field experiment of UScan system operated for two months to collect long-term urban temperature data. To analyze the collected data in an efficient manner, we propose a lightweight clustering methodology to study the correlation between the pattern of temperature and various environmental factors including the amount of sunshine, the width of streets, and the existence of trees. The analysis reveals meaningful results and asserts the necessity of fine-grained deployment of sensors in an urban area.

## 1. Introduction

Since the vision of Smart Dust [1] has been introduced, researchers have explored wireless sensing applications in various fields including healthcare and medical science [2–4], shipping industry [5], environmental monitoring system [6–10]. Although we cannot assert that environmental monitoring is a promising application of wireless sensor networks, its possibility of enhancing our daily lives is highly expected. Thus we are interested in applying a wireless sensor network to support people’s urban life by providing useful information about microclimate in a geographically fine-grained manner. Based on empirical study in this paper, fine granularity of sensors is important for applications of our interest because it increases the possibility of capturing additional and meaningful information. For example, two sensors in our deployment show quite different measured values; nevertheless they are separated by less than three meters. Upon having such fine-grained information in an urban area, one can easily find an ambient walking route, an oasis spot, or a windy and low temperature location in hot summer, for example [11]. In contrast to our work, coarse-grained networks cannot provide detailed information of measured areas. Airy Notes [12] aims to discover the difference of climate between inside and outside of a 583,000-m^2^ park. It cannot capture differences among many spots in the park, e.g., French, English, and Japanese gardens. Also, sparse deployment of CitySense [13] cannot provide as detailed as lane’s or alley’s sensing information. In particular, we cannot acquire air pollutants of an individual alley or water contaminant of a specific rill. Participatory sensing systems such as BikeNet [14], CarTel [15], and [16] extend sensing coverage by allowing sensors go with human; however, we cannot assure availability and/or granularity of sensor data at a certain point. In other words, sensor data at any points are collected intermittently. Hence we cannot collect complete long-term data (for example, a whole week) for further analysis and usage of any applications.

The navigation system developed for pedestrians [11] is one of applications that motivate our work. After installing software into mobile phone, the application acquires temperature data to calculate the most comfortable route. This kind of application requires real-time processing and lightweight computation due to the scarce resource of mobile phone. Fine granularity of sensing is required in order to provide pedestrians with accurate navigation because sensor installed at an opposite side of a building will measure different values of temperature. Fine-grained temperature data are also useful for urban planning. For example, if we know a potential place where heat stroke or heat wave is likely to happen, a city mayor might increase the number of trees and shaded areas by some procedures. Also an electric roof could be installed, and opened or closed automatically according to the current temperature. Regardless of distance between two nearby sensors which are placed under direct sunlight and shaded area, the measured temperature should be quite different. Therefore, it is necessary to deploy a fine-grained wireless sensor network in a city where various factors such as complex infrastructure, miscellaneous roads and streets, tall buildings and skyscrapers, and high population density, affect temperature distribution as well as the flow and strength of wind. The fine-grained sensor network is capable of capturing complexity of environmental information in a city. Hence we have deployed and operated a sensing system called *UScan* by which temperature in fine resolution was measured in downtown Tokyo [17]. The UScan system consists of three main components: a server, wireless relay nodes, and sensor nodes. The server is responsible for collecting temperature data from numerous sensors and managing the database of such sensor data. The wireless relay nodes, which are referred to as wireless bridges, are intermediate nodes that forward received sensing data to the server. To acquire fine-grained sensor data, we installed more than 200 uParts [18] as sensor nodes in 107,500-m^2^ area and collected data for a period of two months during July and August 2007.

After acquiring raw sensor data, the next process is data analysis. Different kinds of sensor data (sound, image, odor, acceleration, *etc.*) have different characteristics; thereby a tailor-made analysis methodology for each data type is preferred [19–23]. Although we collect the same kind of sensor data, each application employs different techniques to interpret and understand the data for specific purposes. Based on a large amount of collected temperature data, we show meaningful experimental results and discuss the correlation between environment factors and the observed temperature by utilizing a proposed clustering methodology. Because the well-known k-means algorithm [24] uses an iterative refinement technique, its processing time is not appropriate for very large database of UScan system. When we utilize sensor data for any real services in an urban area, the amount of data will obviously be larger than that of UScan which is merely a pilot project operated in a small area of downtown Tokyo. Our proposed clustering technique is tailor-made for expediting the calculation process. The environmental factors we are interested include the amount of sunshine, the width of street, and the presence of trees. We extract three features from the variation of temperature data of each day, and utilize such features in the clustering methodology. The proposed clusteringmethod helps understand fine-grained temperature data faster in an efficient manner. The clustering results are able to reveal characteristic of each area or city. By clustering long-term data, we could capture the clustering patterns of an area, and also compare with other areas. Areas whose clustering patterns are similar or different are worth to study in details to reveal the causes of similarity or difference. Urban planning could exploit this kind of information to improve environments of communities by referring to environmental characteristics of favorable communities.

## 2. Related Work

Investigating real-world information in a city is an important task. To achieve such purpose, CitySense [13], which is one of several urban-scale networked sensing systems, has been launched. Sensor nodes are Linux-based embedded PCs outfitted with dual 802.11 a/b/g radios and various sensors for monitoring weather conditions and air pollutants. Nodes are mounted on buildings and streetlights across the city of Cambridge to form a wireless mesh network. The project claimed that it shall consist of approximately 100 nodes in the near future. The system covers wide area but it does not pay attention to fine-grained deployment of sensors. Therefore, the data of complex urban city cannot be acquired by the system.

Airy Notes [12] is an environmental monitoring system that captures temperature, humidity, and acceleration data around sensor nodes. The authors installed 165 sensor nodes in a national park (Shinjuku Gyoen National Garden) which covers 583,000 m^2^ area. The project aims to discover the difference of climate between inside and outside of the park, which can be considered as coarser granularity of sensor network than our work. Also the sensors are deployed in a leisure area, while we focus on urban areas which are highly relevant to and important for our daily lives.

In addition to the above-mentioned works, many kinds of sensor networks have been deployed for studies, experiments, and real-world operations, but most of previous works [6–8, 10, 25, 26] have been installed in environments (garden, forest, lake, ocean, *etc.*) and/or the granularity of installed sensors is not high. Table 1 summarizes node density of previously deployed networks as well as our UScan system. Although we do not have exact deployment area of CitySense [13] and volcano monitoring system [25], it is explicit that node density is lower than that of UScan system.

Recently, researchers are interested in another type of urban sensing based on a concept of participatory or people-centric sensing [27–29]. In this approach, sensors always go with human with his/her daily life, *i.e.*, a kind of mobile sensing system. BikeNet [14] utilizes bicycles mounted with sensors as a mobile sensing system to collect and share ambient data when traveling or commuting. Bicycles are equipped with a Nokia N80 mobile phone, Moteiv Tmote Invent motes, and other necessary sensors. Collected information is exchanged via short-range radios and can be direct (*i.e.*, bike-to-bike) or indirect via the access points which are installed along roads and trails.

Similar to BikeNet, CarTel [15] is a mobile sensor computing system designed to collect data from sensors located on automobiles. A CarTel node is a custom-made device built from a commodityWi-Fi access point with additional enhancements for other sensors. In the presence of opportunistic wireless networks (e.g., Wi-Fi and Bluetooth), each node delivers the sensor readings to a central portal. In addition to managing intermittent connectivity, CarTel provides a simple query-oriented programming interface for the benefit of application developers.

In order to promote people-centric sensing, Ishida *et al.* [16] introduced the concept of *implicit sensing* by using footwear containing pressure sensors. The pressure sensors use the IEEE 802.15.4 radio to send the sensor readings to a client module, which in turn forwards the data to a server via a cellular network. Although the user is equipped with a GPS device, the use of low-cost RFID-based infrastructure has been proposed to determine the location information (indoor and outdoor) corresponding to each sensor reading when GPS signals are not available.

Other mobile sensing systems (Zebranet [9], MetroSense [30], CenceMe [19], *etc.*) have been proposed in the literature. Such systems extend the coverage area of sensing but we cannot assure availability and/or granularity of sensor data at a certain point because mobile sensor nodes are free to move.

## 3. System Architecture and Deployment

In contrast to prior works (e.g., [12, 13]), we deployed finer granularity of sensing system called UScan. In particular, we define a network whose node density is higher than 1,500 nodes/km^2^ as a baseline of fine-grained network. This baseline is much higher than the density of previously deployed networks (see Table 1) which can be considered as coarse-grained networks. This section details system architecture, sensor deployment, and packages of sensors as follows.

### 3.1. UScan System Architecture

**Figure 1.**
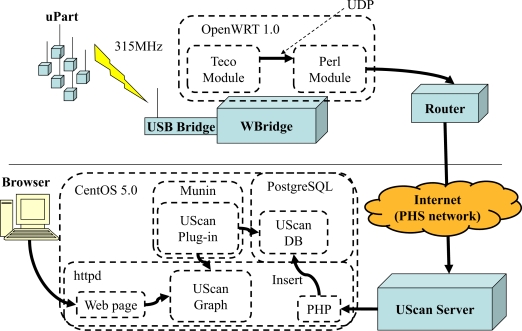
System architecture of UScan.

The system architecture of UScan is shown in Figure 1 [17]. The main components of UScan are a server, wireless bridges (called WBridges), and sensors (called uParts). The uPart [18] sensing devices are responsible for measuring ambient temperature and wirelessly sending data (less than one KB) to the WBridge [31] at a predetermined interval which was set to 30 seconds in our field experiment. The OpenWRT [32], which manages incoming sensor data, is a software installed on the WBridge and consists of Teco and Perl modules. The Teco module transfers the data to the Perl module using a UDP socket. Once the Perl module receives the data, it extracts temperature data and sends them to a UScan server through the Internet. Because the server is set behind NAT (Network Address Translation), the data is sent through port 80 in order to avoid being filtered by firewall. To access the Internet in an outdoor area where wired Internet infrastructure may not be available, we use the Personal Handy-phone System (PHS) which is a mobile network operating in the 1,880–1,930 MHz frequency band. Thus a PHS communication card is attached to each router for this purpose. Although the transmission rate of PHS card (64 kbps) is lower than that of wired infrastructure, its convenience of infrastructure-less connection is an essential requirement to collect data anywhere and anytime. Based on the experiments, 64-kbps transmission rate is high enough to report temperature measurement (less than one-KB packet size) at every 30 seconds. If higher transmission rate is required, 3G cellular network is also available and replacement of PHS card with 3G cellular card is straightforward. Upon receiving the sensor data, the UScan server inserts the data into UScan Database. A Munin Plug-in [33] installed on the server is responsible for monitoring the database and creating a graph as requested by a user through a web API. Currently, users can request temperature graphs by specifying days, time, areas, and sensor IDs.

As mentioned above, we utilize the uPart [18] as a sensor node in our experiment. Although the device is a tiny-sized sensing apparatus, *i.e.,* a dimension of 1 cm by 1 cm by 1 cm, it includes many functions and components such as a wireless communication module, CPU, memory, and many kinds of sensors (illumination, vibration, temperature, and battery’s voltage). It is driven by a button (or coin) cell and can operate for six months (if a packet is sent once every 30 seconds). We choose the uPart because of its light weight and long-life battery which are suitable for long-term environmental monitoring in an urban area. Note that we did not need to replace the battery of all uParts during the entire period (two months) of our experiment. The specification of the uPart is summarized in Table 2.

### 3.2. Fine-Grained Deployment of Sensors

**Figure 2.**
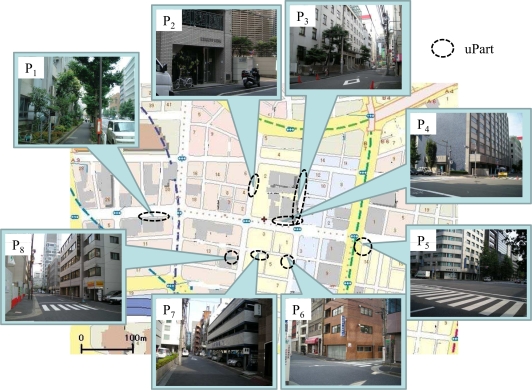
Eight installation points in 250m-by-430m area for 200 sensors.

In an urban area such as downtown Tokyo, there are various environmental factors such as the existence of buildings, parks, and trees that affect the flow of wind and shaded areas which in turn correlate to the variation of temperature. Our policy is to let all observation points cover a wide range of environmental factors that are likely to affect temperature for the benefit of further analysis. However, we have had to negotiate landlords to grant us a permission to install uParts and WBridges, although more than half of landlords refused our requests. In addition, a power supply is necessary for each observation point because WBridge must be in stand-by mode to receive data from uParts, *i.e.*, it cannot switch to sleep mode to minimize energy consumption. We note here that WBridge is able to resume its operation immediately after receiving power in the case of power down or blackout. Finally, we have been granted to deploy approximately 200 uParts in eight observation points which cover a 250m-by-430m area (see Figure 2). As a result, the node density of UScan system is approximately 1,800 nodes/km^2^. To cover various environmental factors, for example, both points *P*_1_ and *P*_5_ locate at pedestrians’ sidewalks along broad streets but they differ in whether shaded areas due to roadside trees exist or not. There are many trees at *P*_1_, while none exists at *P*_5_. Thus each observation point has different environmental factors.

**Figure 3.**
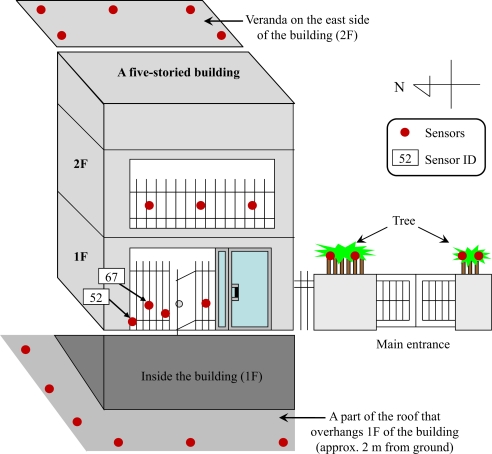
An example of sensor deployment at observation point *P*_2_.

Due to the complexity of urban area, more than 20 uParts were densely installed at each observation point so as to capture possible microscopic characteristics of weather in downtown Tokyo. In particular, we tried to cover all directions (north, east, west, and south) because it is intuitive that temperature measured at the east and west side of a building should be different. Various conditions such as installation on trees, fences, roofs, walls, floors, and verandas are also included as much as possible at any observation points. Even if installation permission has been granted by landlords, we cannot install sensors at any arbitrary points because the landlords asked us to avoid installing sensors in some specific positions due to inappropriate appearance of sensors. In addition, we avoided measuring useless values due to improper installation positions such as nearby exhaust pipes or high-temperature apparatus. Figure 3 shows the detailed sensor deployment of the observation point *P*_2_. The red circles in the figure indicate sensors. Sensors at other observation points were also deployed under the same policy.

### 3.3. Packages of Sensors

**Figure 4.**
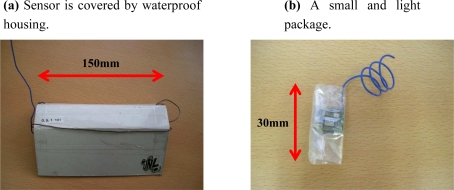
Two types of uPart packages.

The system operates in outdoor areas without human intervention; therefore sensing devices should be able to tolerate various extreme conditions in order to realize fine-grained urban sensing in long period. We developed two types of packages for setting sensors as shown in Figure 4. A package in Figure 4a is used when setting sensors on roadside trees, rain pipes, etc. This type of package is able to shut out direct sunlight and is also waterproof. Temperature sensor is covered with a white roof which is made from waterproof paper. The white color of package helps to reflect the light, *i.e.*, the temperature inside the package is affected the least in comparison with other colors. A simpler one in Figure 4b is used when setting sensors on the wall of building because it is very small and light. Both types of packages are not a closed box, *i.e.*, both left and right sides are open to allow air always flows through the packages. Therefore the sensor inside the package is able to measure correct temperature. However, we have conducted a preliminary experiment to study the effect of the packages to the measured temperature. Based on the preliminary experiment, the temperatures measured by the sensor with the package and the sensor without the package are identical. If there are any effects or differences on the measured temperature, calibration can be easily done.

## 4. Preliminary Results and Investigation

**Figure 5.**
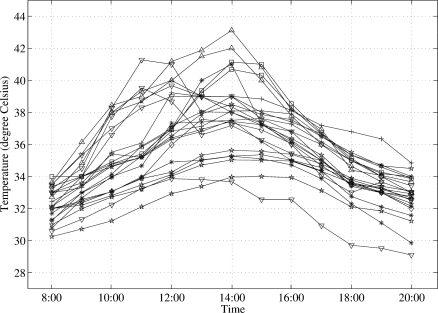
Temperature change measured by 23 sensors placed in a 250m-by-340m area on August 22, 2007.

In this paper, we focus on microclimate during day time because ordinary activities of human life are more active than those of the night time. Thus we use temperature data between 8:00 a.m. and 8:00 p.m. (12 hours each day) for analysis purpose. The median time of the above period is 2:00 p.m. and six hours are available ahead and behind the median time. Figure 5 shows temperature measured by 23 sensors during the day time. We randomly selected 23 sensors from more than 200 available sensors deployed in the 250m-by-340m area so as to ensure that the graph is legible. It is obvious from the figure that the temperatures measured by each sensor at the same time are quite different. In particular, the highest temperature difference is 9 °C at 2:00 p.m. Despite high temperature difference, these 23 sensors located within 500 meters of each other. The underlying reasons of high temperature difference are various environmental factors such as the existence of roadside trees, the width of roads, etc. In addition to temperature difference, there are two distinct patterns of temperature change during the day, *i.e.*, the peak temperature that appears in the morning as opposed to the peak temperature in the afternoon. This is a result of installing sensors on opposite directions (*i.e.*, east versus west). When focusing on microscopic scale, *i.e.*, sensor deployment of observation point *P*_2_ in Figure 3, the temperature difference measured by sensor IDs 52 and 67 which located 10 cm apart is as high as 3 °C on August 22, 2007.

The experiment and preliminary investigation support the necessity of fine-grained deployment of sensors. Note that the data of this field experiment are publicly available at the UScan Website [34].

## 5. Clustering Methodology

To understand the complexity of fine-grained sensor data, an efficient technique to analyze a large amount of collected data is required.

We try to clarify the environmental factors through clustering analysis because clustering divides data into several groups where the characteristics of data in the same group are similar. Based on clustering results, we can further study each group of data in more detail and investigate environmental factors corresponding to each group.

Our clustering methodology is based on three features: bias of temperature, changing rate of temperature, and the maximum temperature of time series temperature data. We plot the results of three features on a 3D-graph where clustering of temperature data is determined. The variables used in the clustering methodology are defined below.

- *D* : the number of observation days.
- *d* : the index of observation days (*d* = 1,..., *D*).
- *M* : the number of observation points.
- *j* : the index of observation points (*j* = 1, ..., *M*).
- *n* : the number of data in one day at each observation point.
- *i* : the index of data in one day (*i* = 1, ..., *n*).
- *k* : the index of features or metrics (*k* = 1, 2, 3).
- *T_dji_* : the temperature data where the observation day is *d*, the observation point is *j*, and the index of data is *i*.
- *f*_*dj*1_ : the bias of temperature data (*i.e.*, the first feature) where the observation day is *d* and the observation point is *j*.
- *f*_*dj*2_ : the changing rate of temperature data (*i.e.*, the second feature) where the observation day is *d* and the observation point is *j*.
- *f*_*dj*3_ : the maximum temperature (*i.e.*, the third feature) where the observation day is *d* and the observation point is *j*.
- *F_djk_* : the normalized value of feature *f_djk_* where *k* = 1, 2, and 3.

Determining features is an essential issue of clustering. We intend to choose three features where their combination is applicable to any seasons as explained below.

### 5.1. Definition of Features

#### 

##### *f*_*dj*1_: Bias of Temperature

The bias of temperature represents the distribution of temperature graph for a given period. The bias is defined as an average of weighted temperature as expressed in Equation (1).

(1) $$f_{\mathrm{dj}1}=\frac{1}{n}\sum_{i=1}^{n}\alpha_{i}\;T_{\mathrm{dji}},\;\;\;\;\text{where}\;\;\alpha_{i}=\{\begin{array}{cc} -\frac{n-1}{2},\ldots ,-2,\;-1,\;0,\;1,\;2,\ldots ,\frac{n-1}{2} & \text{if}\;n\;\text{is odd},\\ -\frac{n-1}{2},\ldots ,-\frac{3}{2},\;-\frac{1}{2},\;\frac{1}{2},\;\frac{3}{2},\ldots ,\frac{n-1}{2} & \text{if}\;n\;\text{is even}. \end{array}$$

The weight *α_i_* is decided by the number of data *n* and the index *i* of time series data. The weight starts from $-\frac{n-1}{2}$ for the first data (*i* = 1) in time series, and increases one for each following data or index. The weight of the last index (*i* = *n*) is explicitly $\frac{n-1}{2}$.

According to the above definition, if the time index is far from the median time (2:00 p.m.) in positive or negative direction (*i.e.*, the right or left direction from the median), the weight *α_i_* will become positively or negatively higher. If the left tail of temperature graph is longer or a temperature graph distorts/bends to the right side, the mass of distribution is concentrated on the right side of the graph and the value of *f*_*dj*1_ is positive—which are referred to as *positive bias*. On the other hand, if the right tail of temperature graph is longer or a temperature graph distorts/bends to the left side, the mass of distribution is concentrated on the left side of the graph and the value of *f*_*dj*1_ is negative—which are referred to as *negative bias*.

The bias is an important feature because it provides the trend of temperature change in a given period. The weight helps to emphasize unclear characteristic of the bias whether it is positive or negative. The value of weight can be adjusted if necessary as long as we use the same definition of weights on the same set of analyzed data. For example, if the bias is not easily noticeable, we may increase the values of weight.

##### *f*_*dj*2_: Changing Rate of Temperature

The changing rate of temperature is defined in Equation (2).

(2) $$f_{\mathrm{dj}2}=\frac{\sum_{i=1}^{n}\left[{\text{max}}_{i}(T_{\mathrm{dji}})-T_{\mathrm{dji}}\right]}{n\;\left[{\text{max}}_{i}(T_{\mathrm{dji}})-{\text{min}}_{i}(T_{\mathrm{dji}})\right]}.$$

The terms max*_i_*(*T_dji_*) and min*_i_*(*T_dji_*) are the maximum and minimum of temperatures observed by sensor *j* in day *d*, respectively. Equation (2) calculates the ratio of temperature difference compared to the maximum to the maximum temperature difference of a day. If we consider temperature graph, in other words, the equation returns the ratio of the area between the maximum temperature and the measured temperature to the entire area of temperature graph.

This feature is an important one because it implies the level of temperature change along a day regardless of average temperature or seasons. The high value of *f*_*dj*2_ indicates radical change of temperature during the day, and vice versa. For example, if the temperature is quite low and steady at 0 °C for a whole day in winter or the temperature is quite high and steady at 30 °C for a whole day in summer, the changing rate of temperature is low. On the other hand, the changing rate of temperature is high, if the temperature varies along a day in spring or fall where average temperature is 15 °C.

##### *f*_*dj*3_: Maximum Temperature

According to Figure 5, the maximum temperatures are different for each observation point. Thus, the maximum value expressed in Equation (3) should be a practical metric when clustering the temperature data.

(3) $$f_{\mathrm{dj}3}=\underset{i}{\text{max}}(T_{\mathrm{dji}}).$$

In addition, the maximum temperature highly relates to outdoor illness such as hyperthermia; thereby it is worth to include it as a feature for clustering purpose.

### 5.2. Normalization

Since the values of each feature have different scales, we normalize the features as expressed in Equation (4).

(4) $$F_{\mathrm{djk}}=\frac{f_{\mathrm{djk}}-{\text{min}}_{\left(d,j\right)}\;(f_{\mathrm{djk}})}{{\text{max}}_{\left(d,j\right)}\;(f_{\mathrm{djk}})-{\text{min}}_{\left(d,j\right)}\;(f_{\mathrm{djk}})},\;\;\text{where}\;k=1,\;2,\;\text{and}\;3.$$

The terms max_(*d,j*)_(*f_djk_*) and min_(*d,j*)_(*f_djk_*) are the maximum and minimum values of feature *k* of all *M* sensors (*j* = 1,..., *M*) for all *D* days (*d* = 1,..., *D*), respectively. After conducting normalization, the range of all features is between zero and one. Thus we can use the normalized features *F_djk_* (where *k* = 1, 2, and 3) in the same space to analyze the complexity of urban environment.

Since the normalized features are relative values of each day, they are applicable to any seasons or weather conditions (e.g., sunny, rainy, cloudy) on the days of interest. Also, we can have meaningful comparison of each day with the help of normalization. Without normalization, we do not know whether a value is high or low in comparison with others.

### 5.3. Definition of Clusters

**Figure 6.**
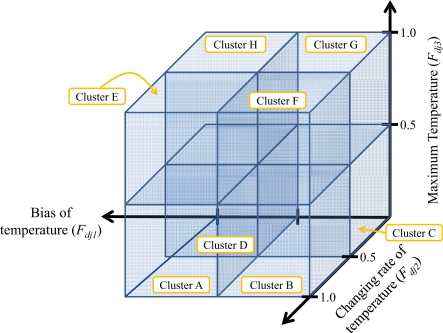
An illustration of eight clusters based on three normalized features.

The normalized bias, changing rate, and maximum of temperature data are plotted on a 3D-graph for clustering purpose. Each feature is divided into two types, *i.e.*, whether a value of feature is higher or lower than a threshold of 0.5. By utilizing three features, there are eight clusters which are referred to as A, B, C, D, E, F, G, and H and illustrated by eight cubes in Figure 6. The first four clusters (A, B, C and D) are allocated to four lower-level cubes (*F*_*dj*3_ < 0.5) in counterclockwise direction. Similarly, the last four clusters (E, F, G and H) are allocated to four upper-level cubes (*F*_*dj*3_ ≥ 0.5) in counterclockwise direction. The definitions of each cluster are detailed below.

Cluster A : *F*_*dj*1_ ≥ 0.5 and *F*_*dj*2_ ≥ 0.5 and *F*_*dj*3_ < 0.5
Cluster B : *F*_*dj*1_ < 0.5 and *F*_*dj*2_ ≥ 0.5 and *F*_*dj*3_ < 0.5
Cluster C : *F*_*dj*1_ < 0.5 and *F*_*dj*2_ < 0.5 and *F*_*dj*3_ < 0.5
Cluster D : *F*_*dj*1_ ≥ 0.5 and *F*_*dj*2_ < 0.5 and *F*_*dj*3_ < 0.5
Cluster E : *F*_*dj*1_ ≥ 0.5 and *F*_*dj*2_ ≥ 0.5 and *F*_*dj*3_ ≥ 0.5
Cluster F : *F*_*dj*1_ < 0.5 and *F*_*dj*2_ ≥ 0.5 and *F*_*dj*3_ ≥ 0.5
Cluster G : *F*_*dj*1_ < 0.5 and *F*_*dj*2_ < 0.5 and *F*_*dj*3_ ≥ 0.5
Cluster H : *F*_*dj*1_ ≥ 0.5 and *F*_*dj*2_ < 0.5 and *F*_*dj*3_ ≥ 0.5

As some other clustering techniques (e.g., k-means and fuzzy c-means clustering algorithms), the number of clusters is an input parameter of the proposed method. An appropriate value, which is a priori unknown, depends on various factors such as the characteristics of data, the number of data, the purpose of clustering, and the clustering algorithm. We could also divide each feature into three ranges equally which leads to 27 clusters in total. Undoubtedly, the data will distribute among 27 clusters and it would be more difficult to capture patterns of any distinctive clusters. Therefore, we decide to use eight clusters and the clustering results in the following section confirm that coarse grain of eight clusters is sufficient for our clustering purpose. Also, a disadvantage of applying finer clustering is higher computational cost.

Specifying the number of clusters a priori is a weakness of our proposed method because an inappropriate choice of number of clusters may yield poor results. As stated in Section 1., this paper focuses on temperature data so that the proposed clustering methodology is designed for temperature data and some features may not be appropriate for other kinds of sensor data. As a result, low adaptability or flexibility of the proposed method is one of possible weaknesses.

## 6. Clustering Results and Comparative Study

This section discusses clustering results and followed by consideration in comparison to the k-means algorithm.

### 6.1. Clustering Results and Analysis

**Figure 7.**
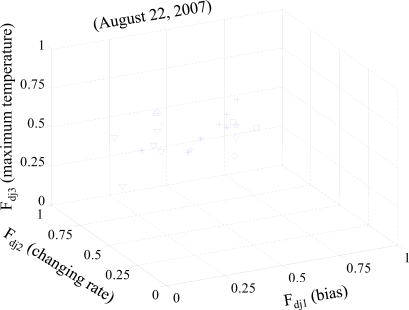
A plot of three features on a 3D-graph (August 22, 2007). Each symbol indicates the sensors being installed under the same environmental factors.

Figure 7 represents three normalized features of temperature data collected on August 22, 2007. There are eight kinds of symbols in the figure where each symbol indicates the sensors being set under the same environmental factors. As one would expect, the same symbols roughly position near each other in the 3D space. We can conclude that the sensors shown by the same symbols detect the same characteristic of temperature on the day of experiment.

**Figure 8.**
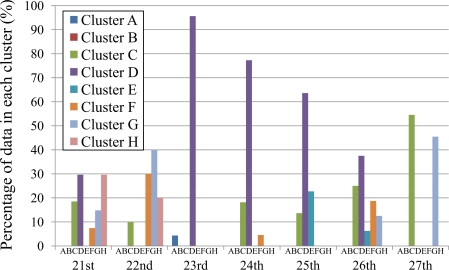
Distribution of temperature in each cluster for the whole week (August 21–27, 2007).

Since the temperature variation differs day by day, we investigate temperature data by considering the distribution of defined clusters on one-day basis for a whole week during August 21–27, 2007. The percentages of sensor data in each cluster of each day are represented in Figure 8. The temperature variation highly depends on the weather condition of each day (sunny, cloudy, *etc.*). Thus we include the period of sunshine in percentage for every two hours from 8:00 a.m. to 8:00 p.m. in Table 3. The data of sunshine period is coarse grain, *i.e.*, they are the percentages of sunshine period in the whole experimental area that covers all of eight installation points. Although the sunshine period over each sensor should be different from the approximate values shown in Table 3, knowing such data is helpful when discussing the clustering results in this section. The data of sunshine period in the table are publicly available at the Japan Meteorological Agency website [35].

In Figure 8, cluster D is apparently distinct on the 23rd, 24th, and 25th where more than half of temperature data (*i.e.,* 96%, 77%, and 63%, respectively) fall under this cluster. The cluster D indicates positive bias (*F*_*dj*1_ ≥ 0.5), low changing rate (*F*_*dj*2_ < 0.5), and low maximum temperature (*F*_*dj*3_ < 0.5). Low amount of sunshine on the 23rd and 24th correlates to two features of cluster D, *i.e.,* low changing rate and low maximum temperature. Although the variation of sunshine does not obviously contribute to positive bias of temperature, the normalized bias of these two days is high enough to cross the border line of 0.5. Merely 4% of data on the 23rd fall under cluster A because of sensors which were installed toward the east and west were affected by the sunshine (sunrise and sunset) and *F*_*dj*2_ of a small amount of sensors are high enough to cross the threshold of 0.5. If the percentage of sunshine is high, more percentage of data should fall under cluster A. The amount of sunshine on the 25th directly leads to positive bias and low changing rate of temperature. However, the amount of sunshine is high on this sunny day; thereby normalized maximum temperature of some data (23%) is above the threshold of 0.5 and falls under other clusters. Therefore, the percentage of cluster D on the 25th (63%) is not so high as those of the 23rd (96%) and 24th (77%).

Although the ratio of cluster D on the 26th (38%) is less than half, it is the most distinct cluster of the day. The underlying reason is that the amount of sunshine is high in the morning in comparison with that of the afternoon. As a result, some of data (25%) show negative bias and fall under cluster C which is the second distinct cluster of the day. Note that the only difference between clusters C and D is the bias of temperature, *i.e,* the features of changing rate and maximum temperature are the same.

The most distinct cluster of the 27th is the cluster C (55%) which indicates negative bias (*F*_*dj*1_ < 0.5), low changing rate (*F*_*dj*2_ < 0.5), and low maximum temperature (*F*_*dj*3_ < 0.5). The variation of sunshine obviously correlates to the properties of negative bias and low changing rate. However, some data show high maximum temperature due to high amount of sunshine in the morning. As a result, 45% of data fall under cluster G, the second-rank cluster of the day, where the only difference in comparison with cluster C is the maximum temperature. We note here that the sensors that were installed toward the east were affected by the sunrise in the morning and the maximum temperature is higher than the threshold of 0.5. If the percentage of sunshine is high all day (both morning and afternoon), the sensors that were installed toward the west should be affected by the sunset in the afternoon and most of data should fall in cluster G.

Cluster G occupies the highest ratio (40%) on the 22nd which is the sunniest day of the week. The result is plausible since cluster G indicates negative bias (*F*_*dj*1_ < 0.5), low changing rate (*F*_*dj*2_ < 0.5), and high maximum temperature (*F*_*dj*3_ ≥ 0.5). Due to the stable amount of sunshine on this day, it is obvious that the maximum temperature should be high and the changing rate of temperature should be low. Also, the 22nd has negative bias because the amount of sunshine in the morning is higher than that of the afternoon.

Two clusters, D and H, equally occupy 30% of the temperature data collected on the 21st. Both clusters indicate positive bias (*F*_*dj*1_ ≥ 0.5) and low changing rate (*F*_*dj*2_ < 0.5), while the characteristic of maximum temperature is different. Cluster D indicates low maximum temperature (*F*_*dj*3_ < 0.5), whereas cluster H shows the opposite one. The amount of sunshine clearly implies positive bias and low changing rate of temperature which are common characteristics of both clusters. It is intuitive that the maximum temperature of each sensor stay around the threshold, *i.e.*, some is above and some is below; thus the temperature data fall under both clusters D and H.

### 6.2. Comparative Study

**Figure 9.**
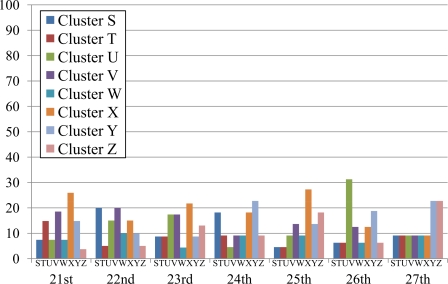
Distribution of temperature in each cluster based on k-means algorithm.

To study how well the proposed methodology presents the characteristics of the clusters, we include the clustering results based on the k-means algorithm in Figure 9 where the number of clusters is set to eight. The eight clusters are named S, T, U, V, W, X, Y, and Z because the definitions of clusters differ from ours. In particular, the definition of cluster is determined by centroids of each cluster which are different on each day. For example, the centroids of each cluster on the 26th are shown in Table 4.

**Figure 10.**
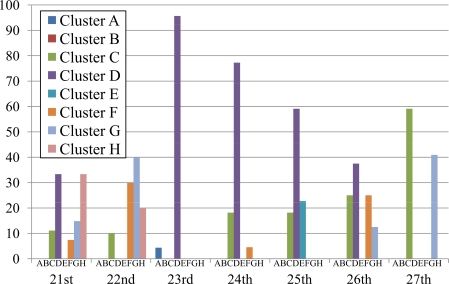
Distribution of temperature after mapping k-means clustering results to the proposed definition of clusters.

It is apparent from Figure 9 that there are no distinctive clusters on each day, *i.e.,* the percentages of each cluster are lower than 30%. As a result, we cannot have any insightful discussion and meaningful information based on these results. Therefore, we decide to map the above results to our definition of clusters (*i.e.*, the clusters A, B, C, D, E, F, G, and H). The centroid of each cluster is used as a criterion to map the whole cluster. For example, cluster S in Table 4 (*F*_*dj*1_ = 0.1835, *F*_*dj*2_ = 0.5297, and *F*_*dj*3_ = 0.5768) falls under cluster F (*F*_*dj*1_ < 0.5, *F*_*dj*2_ ≥ 0.5, and *F*_*dj*3_ ≥ 0.5). Figure 10 shows the results of mapping k-means clusters for the whole week (August 21–27, 2007).

The results of our method (Figure 8) and k-means algorithm (Figure 10) are exactly the same on the 22nd, 23rd, and 24th, while the results are slightly different on the 21st, 25th, 26th, and 27th. However, the trends of clustering results or distinctive clusters are exactly identical. Thus we conclude that our proposed method presents the characteristics of the clusters as well as those of the k-means algorithm.

When considering computational complexity, the proposed clustering technique is linear, *i.e., O*(2*DM*(2*n* + 1)), while the clustering of k-means algorithm [24] can be calculated in exponential time, *i.e., O*(*DM*^*xn*+1^ log*M*), where *x* is the number of clusters. Obviously, the proposed clustering is lightweight and much faster than the k-means algorithm.

## 7. Empirical Analysis on Fine-Grained Data

Previous sections have addressed the correlation between weather and temperature through the results of clustering. In this section, we empirically analyze the fine-grained temperature data by focusing on other environmental factors.

### 7.1. Selection of Representative Data

**Figure 11.**
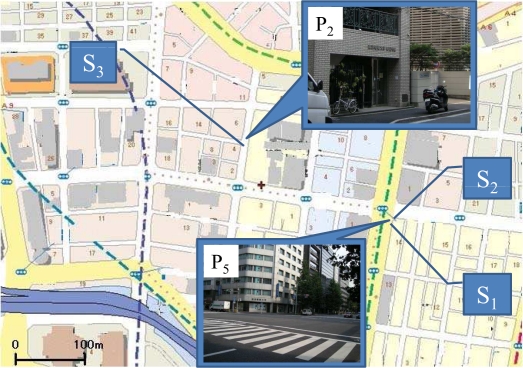
Three observation points *S*_1_, *S*_2_, and *S*_3_.

We selected three days, *i.e.,* the 22nd, 23rd, and 27th, which represent different weather conditions as detailed in Table 5. The 22nd was sunny; the 23rd was rainy; the 27th was sunny in the morning but turned to cloudy in the afternoon. Then we selected three observation points (*S*_1_, *S*_2_, and *S*_3_) with different environmental factors as summarized in Table 6. The environmental factors we are interested include width of street and existence of trees around the installation points of sensors. The width of streets at *S*_1_ and *S*_2_ is six lanes, while *S*_3_ is one-lane street. Trees exist at *S*_1_ and *S*_3_, while none exists at *S*_2_. The locations of three observation points are represented in Figure 11.

### 7.2. Feature-based Distance

We calculate the *feature-based distance* between any arbitrary observation points *S_v_* and *S_w_* by using the definition of Euclidean distance as expressed in Equation (5).

(5) $$\mathrm{Dist}(S_{v},\;S_{w})=\sqrt{{\left(F_{\mathrm{dv}1}-F_{\mathrm{dw}1}\right)}^{2}+{\left(F_{\mathrm{dv}2}-F_{\mathrm{dw}2}\right)}^{2}+{\left(F_{\mathrm{dv}3}-F_{\mathrm{dw}3}\right)}^{2}},$$

where *d* is the observation day.

There are also other definitions of distance to describe how two elements are close to or far away from each other. For example, Mahalanobis distance and normalized Euclidean distance, which are widely used in cluster analysis, take into account the correlations of the data set (*i.e.,* the covariance). In particular, the calculated distance indicates how far a test point is to the center ofmass by also considering the deviation of the data set. As a result, the distance highly depends on the distribution of data set, and it is a useful way of determining similarity of an unknown sample set to a known one.

It is intuitive that similar environmental factors lead to similar pattern of measured temperature. Hence, Mahalanobis distance of an observation point whose environmental factors holds high percentage of data set will be short, and vice versa. In other words, Mahalanobis distance depends on sensor installation of UScan system. Sensors were installed in eight observation points selected from a 250m-by-430m area where the data set may not be large enough to represent the correct distribution of various environmental factors in Tokyo. Therefore, using Mahalanobis distance may not be an appropriate measure because it indicates distance based on the distribution of environmental factors in the limited area. The purpose of calculating feature-based distance is to find relative distance between any two observation points and simple Euclidean distance is able to satisfy the objective.

To refer easily, we define feature-based distances between each of three observation points as follows.

$$u1=\mathrm{Dist}(S_{1},\;S_{2}),\;\;\;\;u2=\mathrm{Dist}(S_{1},\;S_{3}),\;\;u3=\mathrm{Dist}(S_{2},\;S_{3}).$$

### 7.3. Empirical Investigation and Discussion

**Figure 12.**
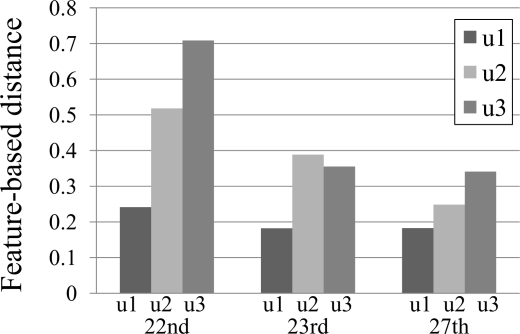
Feature-based distances between selected observation points.

Based on the environmental characteristics of three selected points described in Table 6, u1, u2, and u3 indicate the impact of trees, width of street, and both trees and width of street on temperature change, respectively (see Table 8). The results of feature-based distances (see Figure 12) obviously show that the impact of street width is much higher than that of trees because the distance u2 is longer than u1 on all three days. The values of features used for calculating the distances are given in Table 7. Previous section has showed the correlation between temperature and the amount of sunshine. The result in Figure 12 confirms that the impact of sunshine on temperature also depends on the width of street and the existence of trees, *i.e.,* the distances on sunny day (the 22nd) are the longest.

Figure 12 reveals the difference between two observation points, but we cannot identify the temperature trend of each individual point. With the help of the proposed three features (Table 7), it suggests that *S*_2_, which is a broad street without tree, has the highest maximum temperature with low changing rate (less than the threshold 0.5). This trend is apparent on the sunny 22nd, since temperature highly correlates to the amount of sunshine.

With the exception of the rainy 23rd, the distance u3 is the longest among three distances because it indicates the difference between a six-lane street without tree (*S*_2_) and a one-lane street with trees (*S*_3_). We can conclude that temperature change on a rainy day, which is not affected by sunshine, depends on other factors rather than the width of street and the existence of trees.

The difference of feature-based distance between *S*_1_ and *S*_2_ supports the necessity of fine-grained sensor networks. Both observation points are in very close proximity (see Figure 11). The Euclidean distance between these two points is less than three meters. However, when investigating Table 7, the differences of normalized maximum temperature are approximately 0.15–0.20, *i.e.,* 15%–20% difference.

## 8. Conclusions

In this paper, we have described the system architecture of UScan which is a fine-grained sensor network for studying the characteristic of complex temperature in an urban area. More than 200 sensors have been installed in a 250m-by-430m area in downtown Tokyo, and the temperature data have been continuously collected for two months without any human intervention. The preliminary results in Section 4., where the temperature different of nearby sensors is as high as 9 °C, assert the necessity of fine-grained deployment of sensors in an urban area due to its complexity.

To study the large amount of fine-grained sensor data in an efficient manner, we have proposed a clustering method which is able to classify the variation of temperature and discovered the correlation between temperature change and the amount of sunshine. The clustering results of the proposed method are comparable with those of k-means algorithm, while the propose method enables the cost-effective analysis on very large database without involving high computational cost such as iterative calculations used by the well-known k-means algorithm [24]. In particular, computational complexity of the proposed clustering method is linear, while the k-means algorithm solves the problem of clustering in exponential time. We have further investigated temperature data in fine-grained manners by considering other environmental factors such as the width of street and the existence of trees that also affect temperature change. As a next step, we are planning to study the correlation between temperature and other dynamic factors such as the amount of pedestrians’ and vehicles’ traffic. Traffic information can be obtained by using cameras and pattern recognition techniques [36, 37].

Although fine-grained sensor data provide insightful information in an urban area, we should not deploy sensors too densely because it is not a cost-effective method. However, an appropriate density of sensor deployment depends on both controllable and uncontrollable factors such as deployment environments, target applications, and security concerns. In particular, complicated and unplanned downtown areas require high density of sensors to capture detailed information. High number of redundant sensors is necessary to substitute for malfunctioned sensors in harsh environments. Moreover, an appropriate density is different for each application. Our testbed was deployed for several usages and each node consists of several kinds of sensors (*i.e.,* temperature, vibration, and illumination) which can be utilized for different target applications. When focusing on the scope of the paper where complexity of urban area is an issue, an appropriate density is different for each observation point. To investigate an appropriate value of sensor density by comparing clustering results of multiple node densities, the number of sensors deployed should be higher than an appropriate one which is a priori unknown. However, as mentioned in Section 3.2., the problem of limited installation points hinders us from installing highly dense network to pursue this important issue. As one of our future works, we plan to find more flexible places to perform experiments and investigate the issue of appropriate density.

The computational complexity of both proposed method and k-means algorithm has been analyzed in Section 6.2. Another future work includes further verification by actually measuring the execution times of these both methods because such experiment would show whether the time to cluster the data is significant when the total latency is considered.

As suggested in Section 1., clustering patterns of long-term data could reveal characteristic of each area. To help analyzers to understand data in a more convenient way, we plan to enhance the current web API by letting the analyzers select areas of interest and preferred conditions such as sunny, rainy, or cloudy days to compare clustering results. The analyzers could know, for example, the temperature of which areas change drastically on sunny day. Also, the system could automatically find areas whose clustering patterns are similar as complementary information for the analyzers. If the clustering patterns are similar, we might let some or all sensors of an area sleep temporality so as to prolong the lifetime of sensors. Besides, the analyzers could infer temperature related information from the area where sensors are operating.

By using the UScan data, we will analyze the acquired data in more detail for the purpose of creating efficient fine-grained urban sensing applications. Investigating other kinds of feature as different means of clustering is also our future plan.

## Figures and Tables

**Table 1.** Node density.

**Sensor Deployment**	**Number of Nodes**	**Area (m^2^) or Distance (m)**	**Density (nodes/km^2^)**

UScan	200	107,500 m^2^	1,860
Habitat monitoring [6]	32	959,105 m^2^	33
Airy Notes [12]	165	583,000 m^2^	283
CitySense [13]	100	City of Cambridge	N/A
Volcano monitoring [25]	16	200–400 m apart	N/A

**Table 2.** uPart Specification.

**Dimension**	1 cm × 1 cm × 1 cm
**Sensors**	Temperature, vibration, illumination, and battery’s voltage
**Communication**	Wireless radio (315 MHz)
**Power supply**	A button cell (140 mAh)
**Battery life**	6 months (if transmission interval is 30 seconds)

**Table 3.** Percentages of sunshine period for every two hours during August 21–27, 2007.

	**21st**	**22nd**	**23rd**	**24th**	**25th**	**26th**	**27th**

**8:00**	65%	100%	0%	5%	25%	50%	90%
**10:00**	75%	100%	10%	0%	85%	85%	80%
**12:00**	100%	100%	50%	25%	80%	75%	100%
**14:00**	100%	100%	40%	25%	100%	100%	20%
**16:00**	100%	80%	15%	50%	80%	90%	25%
**18:00**	40%	20%	0%	5%	10%	0%	20%
**20:00**	0%	0%	0%	0%	0%	0%	0%

**Table 4.** Centroids of each cluster on August 26, 2007.

	*F*_*dj*1_	*F*_*dj*2_	*F*_*dj*3_	Mapping results

Cluster S	0.1835	0.5297	0.5768	Cluster F
Cluster T	0.5697	0.0674	0.3116	Cluster D
Cluster U	0.5565	0.2339	0.4509	Cluster D
Cluster V	0.2879	0.2894	0.5128	Cluster G
Cluster W	0.1070	0.7334	0.8436	Cluster F
Cluster X	0.4663	0.5385	0.6404	Cluster F
Cluster Y	0.4130	0.2067	0.3951	Cluster C
Cluster Z	0.4981	0.4268	0.4961	Cluster C

**Table 5.** Weather condition of three selected days.

	**Morning**	**Afternoon**

**22nd**	Sunny	Sunny
**23rd**	Rainy	Rainy
**27th**	Sunny	Cloudy

**Table 6.** Environmental characteristics of three observation points.

**Observation points**	**Width of street**	**Trees**

*S*_1_	Broad	Exist
*S*_2_	Broad	Not exist
*S*_3_	Narrow	Exist

**Table 7.** Three features of three observation points (*S*_1_, *S*_2_, and *S*_3_) in three selected days (the 22nd, 23rd, and 27th).

**22nd**	*F*_(22*nd*)*j*1_	*F*_(22*nd*)*j*2_	*F*_(22*nd*)*j*3_

*S*_1_	0.40	0.22	0.62
*S*_2_	0.45	0.12	0.83
*S*_3_	0.23	0.68	0.46

**23rd**	*F*_(*23rd*)*j*1_	*F*_(*23rd*)*j*2_	*F*_(*23rd*)*j*3_

*S*_1_	0.78	0.47	0.18
*S*_2_	0.80	0.40	0.34
*S*_3_	0.58	0.14	0.24

**27th**	*F*_(27*th*)*j*1_	*F*_(27*th*)*j*2_	*F*_(27*th*)*j*3_

*S*_1_	0.42	0.43	0.38
*S*_2_	0.35	0.33	0.53
*S*_3_	0.34	0.66	0.42

**Table 8.** Environmental factors of interest of each feature-based distance.

**Feature-based distances**	**Environmental factors of interest**

u1 = *Dist*(*S*_1_, *S*_2_)	Trees
u2 = *Dist*(*S*_1_, *S*_3_)	Width of street
u3 = *Dist*(*S*_2_, *S*_3_)	Trees and width of street
